# Bolt Preload Identification Method Based on Multi-Frequency Guided Wave Reconstruction and Spectral Centroid Fusion

**DOI:** 10.3390/s26134184

**Published:** 2026-07-02

**Authors:** Zhangsheng Sun, Zhen Jin, Zhengwu Yi, Haochen Yu, Haishen Zhang, Lining Ma, Xiuquan Li

**Affiliations:** 1 School of Electronic Information and Electrical Engineering, Yangtze University, Jingzhou 434023, China; 2023001627@yangtzeu.edu.cn (Z.S.); 2023001428@yangtzeu.edu.cn (Z.J.);; 2 School of Mechanical Engineering, Yangtze University, Jingzhou 434023, China; 3 School of Future Technology, Yangtze University, Jingzhou 434023, China; 2024000234@yangtzeu.edu.cn

**Keywords:** bolted joints, structural health monitoring, guided wave testing, Chirp excitation, spectral centroid, multi-frequency fusion

## Abstract

Bolted joints are critical load-transfer components in bridges, wind turbines, aerospace systems, mechanical equipment, and offshore platforms, where preload loss can degrade stiffness, accelerate fatigue, and compromise safety. For structural health monitoring, early monitoring of preload reduction before marked loosening is essential, yet existing ultrasonic guided wave indicators remain affected by frequency dependence, non-monotonic responses, amplitude drift, and environmental disturbances. This study proposes an early-warning-oriented preload identification method that combines broadband excitation, multi-frequency narrowband reconstruction, spectral centroid extraction, optimized weighted fusion, and fixed SC-domain linear calibration from one reference loading group. Using a 20–250 kHz Chirp response, 14 narrowband signals from 50 to 180 kHz were reconstructed for an M20 single-bolt specimen tested over 50–90 N·m. The fused spectral centroid index exhibited a stable, monotonic, and approximately linear relationship with preload. When fixed weights and calibration coefficients were transferred to held-out repeated-loading groups, all Pearson correlation coefficients exceeded 0.99. Feature-level robustness tests showed that the arithmetic mean of the spectral centroid reduced temperature-induced *Range%* by 98.42–99.08% and *RSD* by 98.89–99.31% relative to energy-based features. This work provides an interpretable multi-frequency spectral descriptor and a calibration transfer framework for repeatable early warning of preload loss in a controlled single-bolt configuration.

## 1. Introduction

Bolted connections are widely adopted in high-reliability engineering applications such as bridges, wind turbines, aerospace systems, mechanical structures, and offshore platforms due to their structural simplicity, construction flexibility, and cost-effectiveness [1,2,3]. However, during long-term service, bolts are frequently subjected to fatigue loads, impact vibrations, thermal expansion and contraction, and environmental disturbances, leading to preload reduction and even loosening or detachment [4,5,6]. Bolt loosening significantly compromises local structural stiffness and overall load-bearing capacity, triggering fatigue crack propagation, interfacial slippage, or connection failure. In severe cases, this may result in structural collapse, causing engineering disasters and casualties. Consequently, research on efficient, sensitive, and reliable health monitoring methods for bolted connections holds significant importance and practical value in engineering.

From an early-warning perspective, preload monitoring should detect reductions in tightening force before the joint reaches a markedly loosened state. Therefore, quantitative tracking within a warning-oriented preload interval can provide useful information for maintenance decisions before severe loosening or connection failure occurs.

Current bolt condition monitoring techniques primarily fall into two categories:

(1) Direct measurement methods, such as torque wrenches, strain gauges, and load cells, enable quantitative preload estimation. However, they are limited by complex installation, challenges in long-term operation, and sensitivity to external disturbances, making them ill-suited for complex structural field environments [7,8,9];
(2) Indirect assessment methods, including those based on modal frequency [10], acoustic emission [11], piezoelectric impedance spectra [12], vibration response energy [13], or ultrasonic guided waves (GWs) [14,15], infer connection states through signal variations. These approaches offer advantages such as non-contact operation, online capability, and deployable sensor arrays. Among them, ultrasonic GW technology has emerged as a key research focus in recent years due to its sensitivity to local damage, strong penetration capability, and long propagation distance [16,17].

Modern bolt preload monitoring studies also make extensive use of nonlinear ultrasonic features. Contact acoustic nonlinearity (CAN), high harmonic generation, vibro-acoustic modulation (VAM), and wave-mixing-based methods are sensitive to micro-contact changes, frictional slip, partial opening, and weak interface degradation in bolted joints [17,18,19]. These techniques are effective for detecting early-stage loosening because nonlinear wave components can be amplified by imperfect contact interfaces. Their performance, however, is often affected by excitation amplitude drift, sensor coupling variation, and environmental noise, which may reduce their repeatability in long-term field monitoring. The present study focuses on another requirement in bolt health assessment: robust and linearly interpretable preload identification within a relatively tight contact range. For this purpose, the spectral centroid is used as a normalized frequency-domain descriptor. Its parameter-free normalization reduces sensitivity to uniform amplitude variations, while multi-frequency fusion improves its stability against mode conversion, propagation path effects, and temperature disturbances. This provides a complementary route to nonlinear ultrasonic methods for engineering monitoring scenarios where environmental robustness and repeatable quantitative identification are required.

Regarding GW excitation forms, Chirp sweep signals—which are characterized by continuous frequency variation, uniform energy distribution, and precise time-frequency localization—are widely employed in engineering monitoring fields such as radar detection, structural damage identification, and rebar corrosion assessment [20,21,22]. The non-ideal contact condition at bolted interfaces affects guided wave propagation paths, mode conversion, and spectral response characteristics. Under loosened conditions, these changes become more evident in the measured response and its frequency distribution [18,23,24]. Relevant studies demonstrate that Chirp signals effectively excite multi-modal, multi-frequency structural responses. When combined with frequency-domain analysis methods, they significantly enhance bolt condition identification capabilities [25,26,27].

Among frequency-domain feature parameters, the spectral centroid is a representative descriptor that characterizes the center of gravity of the spectral energy distribution. It was first widely used in speech recognition and music analysis [28] and has since been introduced into structural vibration identification, acoustic emission crack localization, fatigue crack growth monitoring, and debonding detection [29,30,31,32]. These studies indicate that the spectral centroid can track shifts in the dominance of high- and low-frequency components and describe the global redistribution of spectral energy, rather than depending only on a single spectral peak or local amplitude value.

Recent bolt monitoring studies have also increasingly adopted machine learning and deep learning methods for tightening and loosening assessment. Fu et al. [33] combined acoustic emission signals with convolutional neural networks and transfer learning for automatic bolt tightness classification. Qin et al. [34] proposed a deep-learning-based method for structural bolt micro-looseness monitoring using batch-normalized stacked autoencoders. Du et al. [35] developed an attention-based multi-task network for temperature compensation in guided-wave-based bolt-loosening identification. These studies demonstrate the ability of data-driven models to extract nonlinear patterns from high-dimensional monitoring data. Their transfer performance, however, remains dependent on the coverage of the training dataset, the structural configuration, the sensor arrangement, and environmental conditions. Therefore, physically interpretable frequency-domain descriptors are still needed for calibration transfer and practical monitoring.

In bolted-joint diagnosis, existing frequency-domain studies more commonly rely on impedance frequency shifts, wave energy transmission, wavelet energy envelopes, transmission coefficients, spectral kurtosis, or spectral amplitude indicators [36,37]. These indicators are sensitive to changes in interfacial contact conditions; however, many are also governed by local spectral variations, signal amplitude, or energy magnitude. Consequently, their responses may be confounded by Lamb-wave dispersion, mode conversion, propagation path diversity, sensor variability, and environmental disturbances [38]. Compared with these frequency-domain indicators, the spectral centroid has rarely been systematically examined as a calibrated preload-sensitive descriptor for bolted-joint diagnosis. Its integration with Chirp-based multi-frequency guided wave reconstruction is even less explored. This methodological gap motivates the present study, in which spectral centroid features are extracted from multiple reconstructed narrowband responses and then fused to enhance monotonicity, repeatability, and robustness within the investigated single-bolt preload conditions.

Furthermore, existing spectral analysis methods predominantly rely on single-frequency excitation or single-point feature extraction, failing to fully exploit the multi-frequency response potential embedded in Chirp sweep signals. This limitation becomes particularly evident under complex interfacial conditions (e.g., sliding, micro-gaps, and disengagement), leading to misjudgments and insufficient sensitivity [19,39]. Spectral centroids or energy features at a single frequency are susceptible to guided wave mode conversion, transducer variability, and propagation path diversity, resulting in non-monotonic indices, large errors, and poor repeatability.

To address these challenges, this study proposes a multi-frequency narrowband pulse reconstruction and spectral centroid fusion method based on Chirp excitation. The approach aims to extract multiple narrowband response signals with distinct center frequencies from sweep responses, calculate spectral centroid parameters for each reconstructed signal, and construct a comprehensive discriminative feature through a weighted fusion strategy. For the present M20 single-bolt specimen, the preload interval of 50–90 N·m was selected as an early-warning-oriented range to track preload reduction before the joint reaches a markedly loosened state. Accordingly, the validation in this study is framed as an assessment of calibration transferability and repeatability within this controlled preload interval and single-bolt configuration. Specifically, the methodology comprises the following key steps:

(1) Chirp Excitation and Response Acquisition: A broad-band Chirp signal spanning 20–250 kHz is employed to excite guided waves in the connected structure, and the corresponding responses are recorded;
(2) Frequency-Domain Filter Construction and Inverse Transformation: Ideal excitation spectra are constructed using Hanning-windowed sinusoidal signals. Frequency-domain filters are subsequently designed to extract responses across multiple bands, enabling the reconstruction of multiple sets of narrowband pulse signals;
(3) Spectral Centroid Calculation and Feature Extraction: The spectral centroid metric is computed for each reconstructed narrowband response signal;
(4) Linear Weighted Fusion Feature Construction: An optimized weighting strategy is applied to enhance the monotonicity of the fused feature relative to preload states;
(5) SC-domain linear relationship evaluation: The calibration transfer behavior of the fused spectral centroid feature is assessed by establishing a fixed linear calibration relationship between the applied preload and the fused spectral centroid response.

Through this multi-frequency fusion mechanism, the proposed method is intended to improve the monotonicity, repeatability, and calibration transfer consistency of spectral-centroid-based preload indicators under the investigated static single-bolt conditions. Therefore, this study should be regarded as a proof-of-concept evaluation of the proposed feature extraction and fusion strategy within a controlled laboratory configuration.

## 2. Detection Principle

### 2.1. Overview of Methodology Workflow

**Figure 1:**
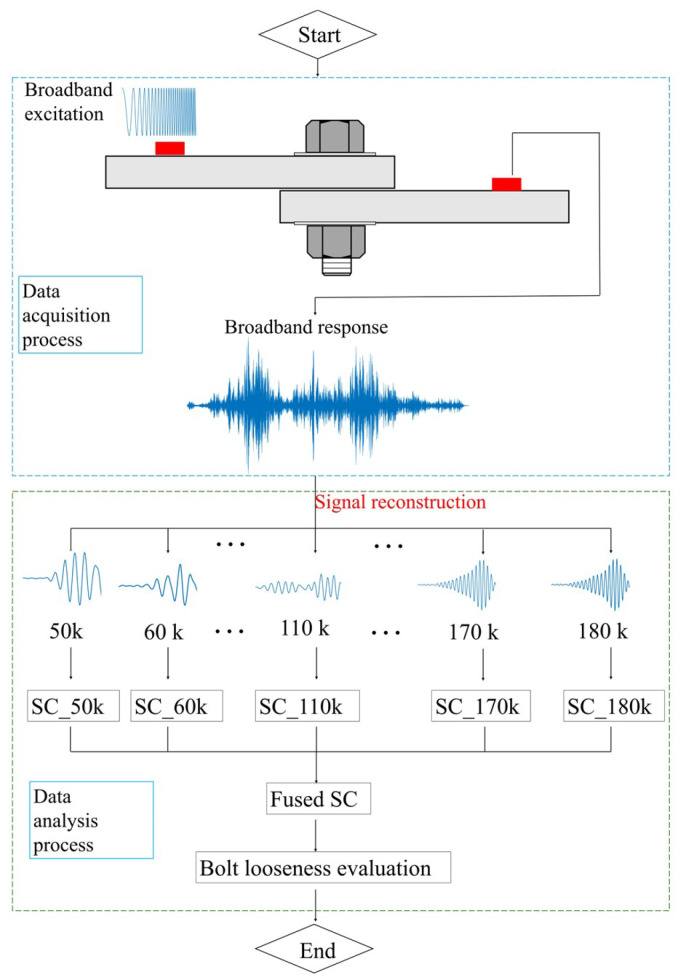
Flowchart of bolt-loosening monitoring method.

The workflow of the proposed bolt looseness monitoring method is illustrated in Figure 1. The process comprises the following key steps: Chirp excitation, response acquisition, frequency-domain filtering, narrowband pulse reconstruction, and the extraction and fusion of spectral centroids.

The filtering used in this reconstruction is a frequency-domain weighting operation rather than a conventional band-pass filter. A Hanning-windowed tone burst is first generated as the target excitation $S_{j}(f)$, and the weighting operator $W_{j}(f)\;=\;S_{j}(f)/S(f)$ is constructed by spectral division with the measured Chirp spectrum, with the division performed at each discrete frequency bin. The reconstructed narrowband response is then obtained through point-wise multiplication, $R_{j}(f)=R(f)⊙W_{j}(f)$, where ⊙ denotes point-wise multiplication, followed by inverse Fourier transformation.

### 2.2. Geometry and Material Properties of the Tested Bolted Joint

Before describing the excitation signal, the geometry and material properties of the tested bolted joint are defined to establish the wave propagation configuration. The specimen consisted of two identical structural steel plates connected by a single M20 high-strength bolt. Each plate measured 300 mm × 60 mm × 10 mm, and the bolt was located at the geometric center of the specimen. This arrangement formed a symmetric single-bolt connection, in which guided waves propagated along the steel plates and interacted with the preload-dependent contact interface in the bolted region.

The plates were made of structural steel with a density of 7850 kg/m^3^, Young’s modulus of 200 GPa, and Poisson’s ratio of 0.30. These geometric and mechanical parameters determine the fundamental waveguide characteristics of the specimen, including wave velocity, modal dispersion, and the contact-dependent transmission response at the bolted interface. After defining the specimen configuration, the Chirp excitation and subsequent frequency-domain reconstruction procedure are introduced in the following section.

### 2.3. Analysis of Chirp Sweep Signal

**Figure 2:**
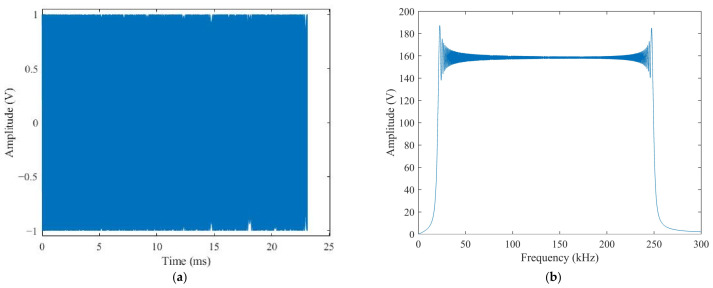
Schematic of frequency sweep signal: (**a**) time-domain representation of Chirp signal; (**b**) Chirp signal spectrum representation.

Figure 2 presents the time-domain waveform and spectral distribution of the employed 20–250 kHz sweep signal. This Chirp signal exhibits continuous frequency distribution and uniform energy, making it well-suited for exciting multi-modal guided wave responses and thus facilitating subsequent frequency-domain reconstruction and feature extraction [40].

### 2.4. Equivalent Frequency-Domain Representation for Signal Reconstruction

In ultrasonic nondestructive testing, the system is typically modeled as a linear time-invariant (LTI) system. The system response r(t) is fundamentally characterized by the temporal convolution of the input signal s(t) and the system’s impulse response h(t):

(1) $$r(t)=s(t)*h(t)=\int_{-\infty }^{+\infty }s(\tau )h(t-\tau )d\tau$$

While the intermediate frequency-domain derivations involving the transfer function H(f) and excitation spectrum S(f) are well established for LTI systems, they provide the theoretical basis for extracting specific narrowband components from broadband responses [41]. By constructing a frequency-domain weighting operator $W_{j}(f_{i})$ from the element-wise ratio between the target narrowband spectrum and the measured Chirp excitation spectrum, the reconstructed time-domain narrowband response $r_{j}(t)$ can be directly obtained through the inverse Fourier transform [40]:

(2) $$W_{j}(f_{i})=\frac{S_{j}(f_{i})}{S(f_{i})},\;\;\;\;r_{j}(t)=F^{-1}[R(f)⊙W_{j}(f)]$$

where $f_{i}$ denotes the i-th discrete frequency bin, $R(f_{i})$ denotes the measured response spectrum, ⊙ denotes element-wise multiplication, and $F^{-1}$ represents the inverse Fourier transform operator. This notation indicates that both the spectral division used to construct $W_{j}(f_{i})$ and the multiplication used for reconstruction are performed point by point in the frequency domain.

### 2.5. Frequency Dependence of Multi-Frequency Narrowband Guided Wave Responses

In the present work, the LTI formulation is adopted as an equivalent reconstruction model for feature extraction within the experimentally investigated preload range (50–90 N·m). Its applicability to more severely loosened states, where stronger contact acoustic nonlinearity may arise, is beyond the scope of this study.

**Figure 3:**
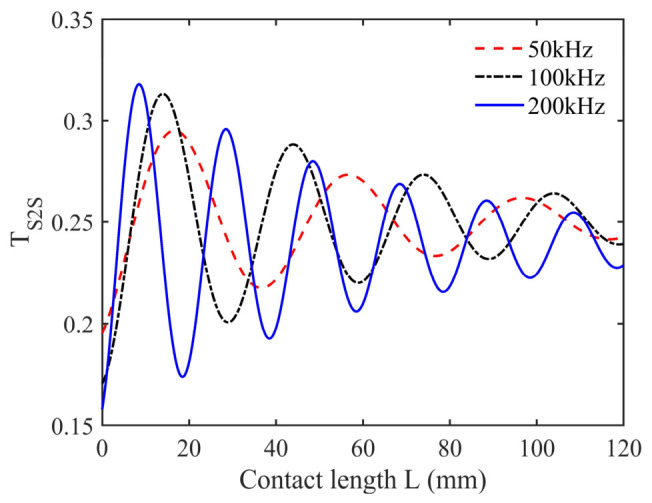
Transmission coefficients of narrowband guided waves at different center frequencies.

Previous studies have shown that energy-based metrics under single frequency excitation are affected by Lamb wave dispersion, mode conversion, sensor variability, and propagation path effects, which may cause modal aliasing and unstable identification results. As reported in our previous work [20], the guided wave transmission coefficient $T_{s2s}$ changes with contact length in a frequency dependent and non-monotonic manner. Here, $T_{s2s}$ denotes the S0-to-S0 modal transmission energy ratio, namely the proportion of incident S0-mode guided wave energy that remains in the S0 mode after transmission through the bolted joint interface. This coefficient describes the energy coupling efficiency of the S0 mode across the contact interface, and its definition follows the modal decoupling calculation method reported in Ref. [20]. Figure 3 was redrawn by the authors based on the frequency-dependent transmission behavior reported in Ref. [20] to illustrate the S0 mode response at different center frequencies. The different variation patterns at 50, 100, and 200 kHz indicate that individual frequency bands may have different sensitivities to interface state variations.

Consequently, this study employs a multi-frequency narrowband pulse reconstruction method based on Chirp encoding to extract frequency-selective narrowband response signals across multiple frequency bands. This approach enhances the sensitivity and robustness of bolted connection condition identification.

### 2.6. Definition of Spectral Centroid and Analysis of Robustness Advantages

To characterize the structural response at specific frequencies, spectral analysis was performed on the reconstructed narrowband guided wave signals. The spectral centroid (SC) was selected as the primary feature parameter. The SC represents the energy-weighted center of the frequency distribution and describes the central tendency of the spectral energy.

Because the experimentally acquired signals are discrete time series, all Fourier transforms and feature calculations in this study were performed in the discrete domain. For the j-th reconstructed response $r_{j}[n]$, the discrete spectrum was obtained using the fast Fourier transform (FFT):

(3) $$X_{j}(f_{i})=FFT[r_{j}[n]]$$

where $f_{i}$ denotes the i-th frequency point, $X_{j}(f_{i})$ is the corresponding spectral amplitude, and N is the total number of frequency points considered in the calculation. The spectral centroid of the j-th reconstructed response was then calculated as follows:

(4) $$SC_{j}=\frac{\sum_{i=1}^{N}f_{i}\cdot |X_{j}(f_{i}){|}^{2}}{\sum_{i=1}^{N}|X_{j}(f_{i}){|}^{2}}$$

This parameter describes the redistribution of spectral energy caused by variations in the bolted-interface state. It therefore provides a compact frequency-domain descriptor of the reconstructed guided wave response.

For comparison, the signal energy $E_{j}$, which is commonly used to characterize guided wave intensity, was calculated as follows:

(5) $$E_{j}=\sum_{i=1}^{N}|X_{j}(f_{i}){|}^{2}$$

Energy-based features depend directly on the squared spectral amplitude. They are therefore sensitive to excitation amplitude, sensor coupling, and other non-structural variations during experiments. In conventional ultrasonic testing, such amplitude disturbances may arise from coupling changes, excitation inconsistency, or environmental fluctuations, which can reduce the stability of energy-based condition indicators [42].

In this study, the spectral centroid was used as a robust alternative. As an energy-weighted average frequency, it includes a normalization term in the denominator and is therefore insensitive to uniform amplitude scaling of the entire spectrum [43]. Rather than measuring absolute acoustic intensity, the SC describes the state-dependent redistribution of spectral energy within the reconstructed response. By tracking the overall spectral tendency, it can capture bolt-loosening-induced spectral changes while reducing the influence of local amplitude fluctuations. Previous studies in structural health monitoring and acoustic nondestructive testing have also reported that spectral-centroid-based features exhibit good stability and repeatability under excitation and temperature disturbances [42,43,44].

### 2.7. Multi-Frequency Spectral Centroid Fusion Methodology

To address the inherent variability of single-frequency metrics and construct a discriminative feature with enhanced robustness to complex preload states, this study proposes two spectral centroid fusion strategies.

#### 2.7.1. Arithmetic Mean Fusion

The fused spectral centroid ${SC}_{avg}$ is formed by averaging the individual spectral centroids extracted from multiple reconstructed frequency bands. This approach simplifies the characterization of overall spectral response trends. The calculation formula is as follows:

(6) $$SC_{\mathrm{avg}}=\frac{1}{M}\sum_{j=1}^{M}{SC}_{j}$$

where: *M* = number of reconstructed pulse responses, ${SC}_{avg}$ = fused spectral centroid, and ${SC}_{j}$ = spectral centroid corresponding to the j-th frequency.

This strategy achieves a simplified characterization of global frequency trends but does not fully account for response variations across frequency bands.

#### 2.7.2. Linear Weighted Fusion Method

To enhance the monotonicity and sensitivity of the fused feature to preload variations, an optimized weighting strategy is introduced.

Construction of the weight optimization objective: To prevent information leakage between calibration and validation, the fusion weights are determined exclusively from the reference loading group in each validation fold. For reference loading group *r*, the spectral centroid matrix is expressed as follows:

(7) $$S^{\left(r\right)}=\left[\begin{array}{cccc} SC_{1}^{\left(1,r\right)} & SC_{2}^{\left(1,r\right)} & \cdots & SC_{M}^{\left(1,r\right)}\\ SC_{1}^{\left(2,r\right)} & SC_{2}^{\left(2,r\right)} & \cdots & SC_{M}^{\left(2,r\right)}\\ \vdots & \vdots & \ddots & \vdots \\ SC_{1}^{\left(N_{cal},r\right)} & SC_{2}^{\left(N_{cal},r\right)} & \cdots & SC_{M}^{\left(N_{cal},r\right)} \end{array}\right]$$

where M is the number of reconstructed frequency bands, and $N_{cal}$ is the number of calibration preload levels. The corresponding fusion weight vector is defined as follows:

$$w^{\left(r\right)}=[w_{1}^{\left(r\right)},w_{2}^{\left(r\right)},\cdots ,w_{M}^{\left(r\right)}]$$

The weight vector is obtained by minimizing the deviation between the fused spectral centroid and the calibration preload trend within the reference group:

(8) $$w^{\left(r\right)}=\arg\underset{w}{min}{\left\|S^{\left(r\right)}w^{T}-q^{\left(r\right)}\right\|}_{2}^{2}+\lambda {\left\|w-w_{0}\right\|}_{2}^{2}$$

where $q^{\left(r\right)}$ denotes a prescribed normalized preload trend vector constructed only from the applied torque levels in the reference group. It is defined element-wise as follows:

(9) $$q_{k}^{\left(r\right)}=\frac{T_{k}-T_{min}}{T_{max}-T_{min}},\;\;k=1,2,\ldots ,N_{cal}$$

where $T_{k}$ is the applied torque at the k-th calibration preload level, while $T_{min}$ and $T_{max}$ are the lower and upper bounds of the calibration preload range, respectively.

Thus, $q^{\left(r\right)}$ is a normalized linear ramp determined solely by the commanded preload levels. It is entirely independent of the measured spectral centroid data and does not rely on empirical spectral centroid values at the extreme preload levels. The parameter λ is a regularization coefficient, and $w_{0}=[1/M,1/M,\cdots ,1/M]$ represents the arithmetic mean fusion vector.

The regularization term penalizes excessive deviations from the mean fusion baseline, thereby reducing the risk of overfitting when only a limited number of calibration preload levels are available. The optimization problem is subject to the following constraints:

(10) $$\sum_{j=1}^{M}w_{j}^{\left(r\right)}=1,\;\;0\leq w_{j}^{\left(r\right)}\leq 1$$

After the fusion weight vector $w^{\left(r\right)}$ is obtained from the reference loading group, it is kept fixed and used to integrate the spectral centroid features extracted from the remaining validation groups. For a validation sample or loading group with an unknown preload, the weighted fused spectral centroid is calculated as follows:

(11) $$SC_{fused}=X_{test}w^{\left(r\right)}$$

where $X_{test}$ denotes the spectral centroid matrix of the tested sample or loading group. The weighted fused spectral centroid $SC_{fused}$ is a pre-calibration frequency-domain feature calculated only from the measured guided wave response and the weight vector derived from the reference loading group. Because each individual spectral centroid is computed along the FFT frequency axis, $SC_{fused}$ is expressed in Hz when the frequency axis is defined in Hz.

The relationship between $SC_{fused}$ and the applied preload can first be evaluated directly through monotonicity and correlation analyses. To further quantify the linear calibration transfer behavior of this feature, a fixed SC-domain linear calibration model between the applied torque and the fused spectral centroid is established using only the reference loading group:

(12) $${\hat{SC}}_{fused,i}^{lin,(r)}=\alpha^{\left(r\right)}T_{i}+\beta^{\left(r\right)}$$

where $T_{i}$ denotes the applied torque at the i-th calibration preload level, ${\hat{SC}}_{fused,i}^{lin,(r)}$ denotes the linearly fitted fused spectral centroid value for reference loading group r, and $\beta^{\left(r\right)}$ and $\alpha^{\left(r\right)}$ are the corresponding slope and intercept calibration coefficients, respectively. Accordingly, the units of $\alpha^{\left(r\right)}$ and $\beta^{\left(r\right)}$ are Hz/(N·m) and Hz, respectively.

The calibration coefficients are determined by least-squares fitting using only the reference loading group:

(13) $$\left(\alpha^{\left(r\right)},\beta^{\left(r\right)}\right)=\arg\underset{\alpha ,\beta }{min}\sum_{i=1}^{K_{cal}}{\left(SC_{fused,i}^{\left(r\right)}-\alpha T_{i}-\beta \right)}^{2}$$

where $K_{cal}$ denotes the number of calibration preload levels, and $SC_{fused,i}^{\left(r\right)}$ denotes the observed fused spectral centroid at the i-th calibration preload level in the reference loading group. Once $\alpha^{\left(r\right)}$, $\beta^{\left(r\right)}$, and $w^{\left(r\right)}$ are determined from the reference group, they remain fixed for all held-out validation groups. Accordingly, the validation samples are processed using only their measured guided wave responses and the fixed parameters derived from the reference group. No preload-level-dependent correction or scaling factor is introduced during validation. The residual metrics were calculated in the fused-spectral centroid domain and are therefore expressed in Hz.

### 2.8. Performance Evaluation Metrics

To evaluate the transfer performance of the fixed fusion and SC-domain linear calibration model described in Section 2.7.2, the validation groups were assessed using three metrics: root mean square error in the fused-spectral centroid domain, Pearson correlation coefficient, and maximum error in the fused-spectral centroid domain. The $RMSE_{SC}$ and $MaxE_{SC}$ values were calculated from the residuals between the observed fused spectral centroid values and the corresponding linearly fitted fused spectral centroid values obtained using the fixed reference group calibration relationship. Therefore, these two residual metrics are expressed in Hz. The Pearson correlation coefficient (R) was used to quantify the linear association between the fused spectral centroid index and the applied torque.

The root mean square error in the fused-spectral centroid domain is as follows:

(14) $$RMSE_{SC}=\sqrt{\frac{1}{n}\sum_{i=1}^{n}{\left(SC_{fused,i}-{\hat{SC}}_{fused,i}^{lin}\right)}^{2}}$$

The Pearson correlation coefficient (R) is as follows:

(15) $$R=\frac{n\sum \left(x_{i}y_{i}\right)-\sum x_{i}\sum y_{i}}{\sqrt{\left[n{\sum {x_{i}}^{2}-\left(\sum x_{i}\right)}^{2}\right]\left[n{\sum {y_{i}}^{2}-\left(\sum y_{i}\right)}^{2}\right]}}$$

The maximum error in the fused-spectral centroid domain is as follows:

(16) $$MaxE_{SC}=\underset{i}{\max}\left|SC_{fused,i}-{\hat{SC}}_{fused,i}^{lin}\right|$$

where n denotes the number of validation samples, $SC_{fused,i}$ is the observed fused spectral centroid value of the i-th validation sample, and ${\hat{SC}}_{fused,i}^{lin}$ is the corresponding linearly fitted fused spectral centroid value calculated using the fixed SC-domain linear calibration relationship. Both quantities are expressed in Hz. In Equation (15), $x_{i}$ denotes the applied torque, and $y_{i}$ denotes the fused spectral centroid index. This evaluation assesses whether the fusion weights and SC-domain linear calibration relationship derived from the reference group can be transferred to independent repeated-loading datasets acquired under the same predefined preload levels.

## 3. Experimental Validation

To verify the effectiveness and robustness of the proposed bolt preload identification method based on multi-frequency reconstruction and spectral centroid fusion, a structural guided wave testing platform was designed and constructed. Comparative experiments were conducted under repeated loading and controlled disturbance conditions. The experimental scope included the comparison of guided wave responses across different frequencies, extraction of spectral centroid features, evaluation of calibration transferability across repeated loading groups, and feature-level assessment of spectral centroid stability under variations in excitation amplitude, structural length, and temperature.

**Figure 4:**
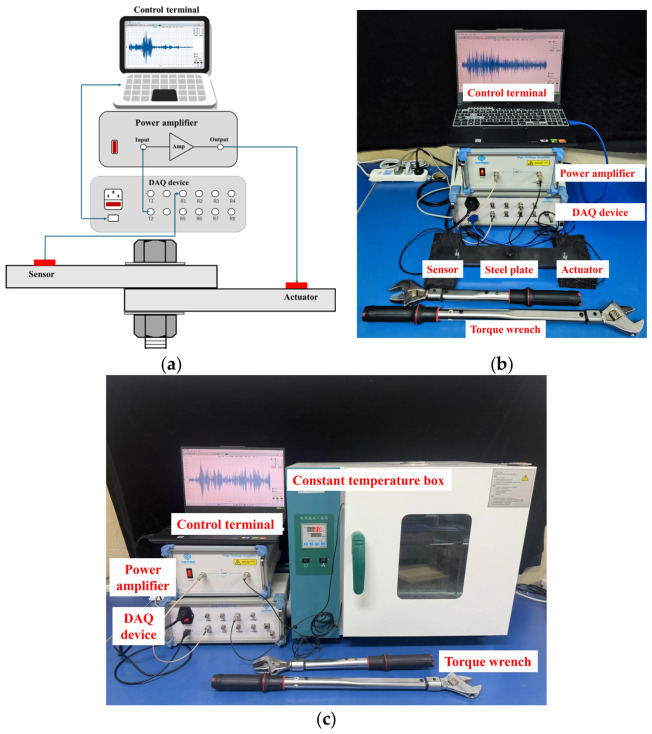
Bolt preload monitoring system: (**a**) Schematic of the experimental setup; (**b**) laboratory setup; (**c**) temperature experiment site diagram.

The specimen comprised two steel plates (300 mm × 60 mm × 10 mm) connected by an M 20 high-strength bolt. The structural configuration is shown in Figure 4a and comprises custom-built ultrasonic transducers, a data acquisition (DAQ) system, a power amplifier (gain = 25), and a laptop equipped with DAQ control software(V1.0). The physical setup is presented in Figure 4b. Each ultrasonic transducer was fabricated using a circular PZT wafer with a diameter of 12 mm and a thickness of 2 mm. The PZT element was housed in a stainless-steel shell to provide mechanical protection and electromagnetic shielding. A magnet was bonded to the back side of the PZT wafer, allowing for convenient attachment to the steel plate and acting as a backing mass to improve guided wave energy transfer. The fabricated transducer had an overall height of 10 mm and an outer diameter of 16 mm.

The data acquisition system was based on an eight channel SC-HY-PZT-2.0 DAQ board (Sanchuan Inc., Jiangsu, China), which was used for waveform generation and signal acquisition. A linear Chirp excitation signal spanning 20 to 250 kHz was generated by the DAQ system, amplified by the power amplifier, and then applied to the excitation transducer to generate broadband Lamb waves in the bolted plate. The Chirp excitation amplitude was set to 4 V before amplification, and the excitation duration was 0.231 s. The response signal was recorded by the receiving transducer through the acquisition module of the DAQ system and saved on the laptop for subsequent processing. The sampling frequency was set to 2 MHz, and the acquisition duration was 0.25 s to ensure the complete recording of the broadband guided wave response.

Preload setting: To evaluate preload identification within a controlled and tightly coupled contact range, the applied torque was varied from 50 to 90 N·m in increments of 5 N·m. This interval was selected as an early-warning-oriented preload range for the tested M20 single-bolt joint. In the present laboratory configuration, torque levels above 90 N·m corresponded to a tightly clamped contact condition, whereas torque levels below 50 N·m indicated a markedly loosened state. Therefore, this study focused on quantitative preload tracking within the 50–90 N·m interval, where variations in the bolted contact condition are practically meaningful for preload-loss warning in structural health monitoring. At each torque level, three independent datasets were acquired to evaluate repeatability and to support reference-group-based validation.

Torque control accuracy and uncertainty: The bolt torque was applied using a calibrated torque wrench. Within the torque range relevant to this study, the wrench repeatability was 0.1 N·m at all calibration points, and the expanded uncertainty was 1.0% with k = 2. The actual torque indication error over the investigated range of 50–90 N·m was −0.30 to −0.70 N·m, with the maximum absolute error occurring at 90 N·m. Because the experimental torque interval was 5 N·m, both the torque control uncertainty and the indication error were smaller than the loading interval and were therefore considered acceptable for the present controlled preload-monitoring validation.

Validation design: Three independent repeated-loading datasets were acquired at each preload level, and a reference-group-based validation strategy was adopted. For each fold, one loading group was selected as the reference group to determine the multi-frequency fusion weights and the global SC-domain linear calibration coefficients. The remaining two loading groups were excluded from both weight optimization and calibration and were used exclusively for the held-out validation. After the fusion weights and calibration coefficients were fixed, the held-out guided wave responses were used without torque labels to calculate the fused spectral centroid features. The applied torque labels were introduced only afterward to compute the correlation and SC-domain residual metrics. Therefore, this protocol assesses calibration transferability and repeatability across repeated loading groups within the same single-bolt configuration and the investigated preload interval of 50–90 N·m. The results should therefore be interpreted within this controlled validation scope and should not be extended to unseen torque levels, different joint geometries, dynamic loading conditions, or unknown field operating conditions.

The Chirp signal parameters are shown in Table 1: the frequency range covers multiple guided wave modal excitation bands, enabling subsequent multi-frequency reconstruction. The acquired broadband responses were processed via frequency-domain filtering and inverse transformation to extract 14 equivalent narrowband pulse responses with center frequencies distributed between 50 and 180 kHz. These signals were used for spectral centroid extraction and fusion.

Additionally, experiments involving structural-length variations and excitation amplitude changes were conducted to evaluate the feature-level stability of the spectral centroid descriptor under controlled disturbance conditions. Steel plates of different lengths (180 mm, 225 mm, and 300 mm) were tested under excitation voltages ranging from 3.0 to 5.0 V across multiple loading cycles. All tests maintained consistent bolt specifications, transducer placement, and loading procedures, with only a single variable adjusted to isolate influencing factors. This design facilitates the analysis of spectral feature variations under structural parameter changes and excitation disturbances. The experimental results are detailed in Section 4.3.

Furthermore, to assess the robustness of spectral centroid features against environmental variations, temperature perturbation experiments were performed using a climate chamber. Bolt preload conditions were fixed at 50, 60, and 70 N·m, while guided wave responses were recorded during stepwise temperature increases. This setup evaluates the impact of temperature on spectral feature stability. The temperature-controlled experimental apparatus is shown in Figure 4c, with analysis results presented in Section 4.4.

## 4. Results

### 4.1. Validation of Narrowband Pulse Reconstruction Effectiveness

To verify the applicability and effectiveness of the proposed frequency-domain reconstruction method, comparative experiments were conducted on a 300 mm steel plate under a 70 N·m preload condition. The control method employed traditional single-frequency excitation using a Hanning-windowed five-cycle tone burst as the excitation signal. This waveform offers concentrated main-lobe energy and rapid side-lobe attenuation, effectively suppressing adjacent-frequency interference while highlighting target frequency characteristics.

**Figure 5:**
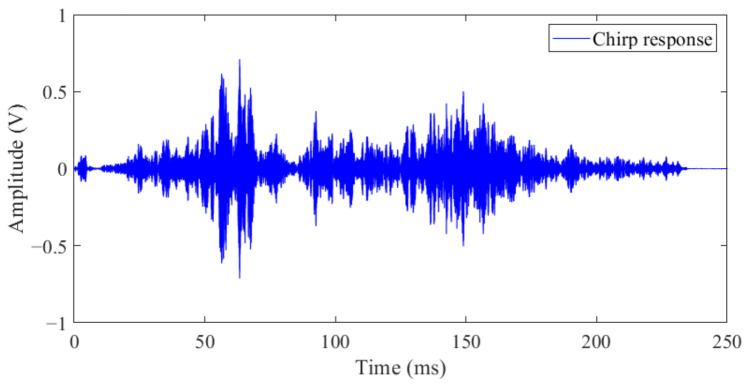
Response of Chirp-encoded signal.

Figure 5 displays the acquired Chirp response. Based on this broadband response, the frequency-domain reconstruction strategy was applied to extract narrowband response signals at different center frequencies.

**Figure 6:**
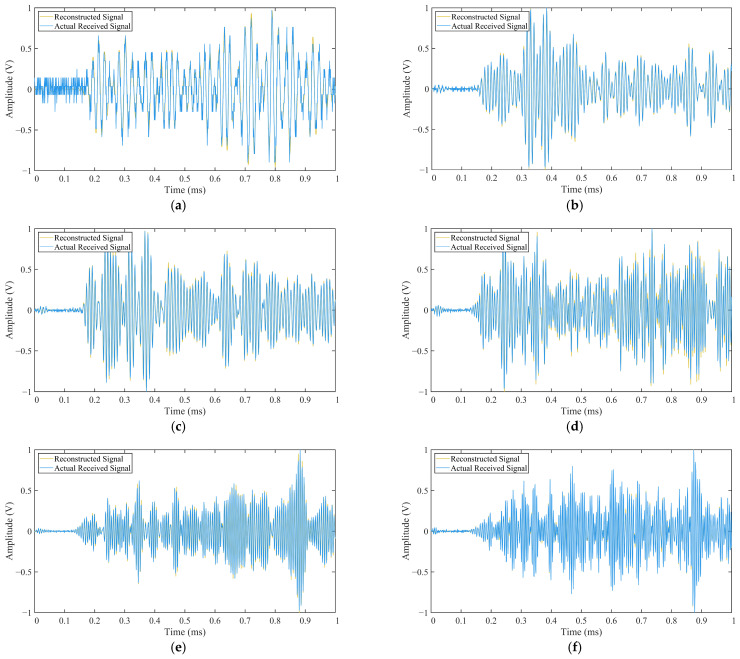
Schematic diagram of comparison between actual received signals and reconstructed signals at different frequencies: (**a**) 50 kHz; (**b**) 80 kHz; (**c**) 100 kHz; (**d**) 120 kHz; (**e**) 150 kHz; (**f**) 180 kHz.

To provide both visual and quantitative validation of the reconstruction accuracy, the reconstructed signals were compared with the signals actually received and obtained under direct narrowband excitation, as summarized in Table 2 and illustrated in Figure 6. The results show that the reconstructed and measured signals maintain high waveform similarity and Pearson correlation coefficients across the selected frequencies, with low normalized mean square error (NMSE) values, thereby confirming the effectiveness of the proposed frequency-domain reconstruction method.

In particular, the waveform similarity and Pearson correlation coefficient exceed 97% at all reported frequencies and approach 99.8% for 80–180 kHz, while the NMSE remains below 5% in all cases and below 0.5% for most of the selected frequencies.

The comparison demonstrates that the reconstructed responses outperform directly acquired signals in waveform fidelity, amplitude stability, and signal-to-noise ratio (SNR). They exhibit enhanced frequency-focusing capability and superior noise immunity. These results support the validity of the proposed reconstruction method based on swept-sine system function decomposition, which significantly improves signal quality and feature extraction stability while preserving frequency resolution.

It should be noted that, although division by a very small excitation spectrum may in principle amplify noise, the frequency band adopted for subsequent analysis (50–180 kHz) was selected because it provided adequate reconstruction accuracy and favorable energy-coupling efficiency under the present experimental conditions. In particular, the comparison at 80 kHz in Figure 6b, corresponding to a relatively low-amplitude region of the Chirp spectrum, shows that the reconstructed response still agrees well with the directly measured signal and does not exhibit significant noise amplification within the investigated SNR range.

### 4.2. Comparative Analysis of Single-Frequency Spectral Centroids

In this study, the Chirp excitation spanned 20–250 kHz. Based on the established system function model, narrow-band pulse responses within 50–180 kHz were extracted at 10 kHz intervals. This frequency band exhibits a high signal reconstruction accuracy and favorable energy coupling efficiency, making it suitable for frequency-domain feature extraction. Data were acquired from 300 mm steel plates under ambient temperature conditions.

For figures containing error bars, each marker represents the mean value of three repeated measurements under the same test condition, and the error bar denotes ± one sample standard deviation calculated from these three measurements.

**Figure 7:**
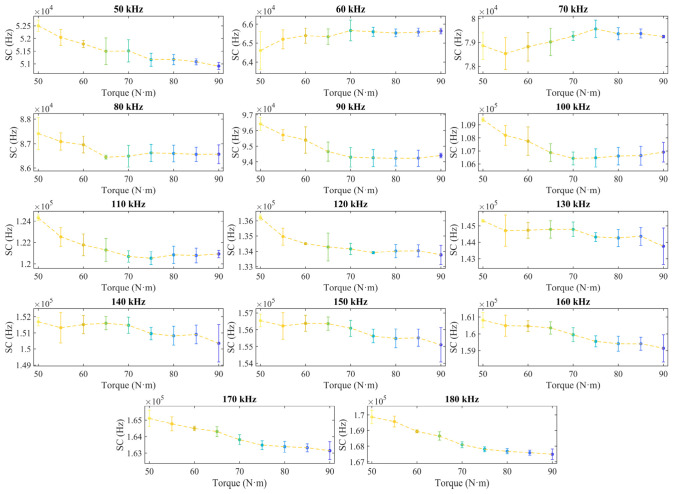
Spectral centroids (SCs) at different frequencies across three loading cycles.

Figure 7 illustrates the variation in spectral centroids (SCs) with bolt preload across different reconstructed frequencies. Key observations include the following:

(1) At 50 kHz and 60 kHz, the SCs demonstrate significant monotonic trends with increasing preload;
(2) Within 70–150 kHz, the SCs exhibit pronounced fluctuations and non-monotonic behavior;
(3) At 160 kHz and 180 kHz, the SCs show a stable monotonic decrease with increasing torque.

This phenomenon indicates that the contact state at the bolted interface imposes a frequency-selective influence on guided wave propagation responses. It reflects varying sensitivities of different frequencies to contact nonlinearity. Consequently, single-frequency features fail to reliably reflect loosening severity, necessitating the adoption of multi-frequency fusion strategies to enhance identification reliability and robustness.

To enhance the stability and anti-interference capability of bolt preload identification features, this study implements fusion processing on spectral centroid metrics extracted across multiple frequency bands. Two strategies are introduced: arithmetic mean fusion and linear weighted fusion.

**Figure 8:**
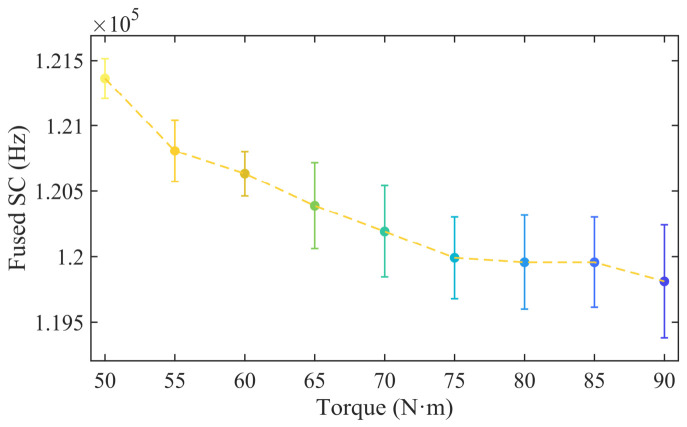
Spectral centroids (SC) based on arithmetic mean.

Figure 8 illustrates the variation trend of the fused spectral centroid using the arithmetic mean strategy. The results exhibit an overall monotonic decline with increasing torque, partially mitigating the detection deficiency observed at specific frequency bands during early-stage loosening. Nevertheless, this approach fails to account for the susceptibility differences of frequency components to damage, resulting in the limited linearity of the fusion results [45].

To evaluate the calibration transfer behavior of the proposed multi-frequency fusion strategy, a reference-group-based validation protocol was adopted. In each fold, the fusion weight vector and the global SC-domain linear calibration coefficients were determined exclusively from one reference loading group and then kept fixed. These fixed parameters were subsequently applied to the remaining two repeated loading groups for held-out validation. Prior to SC-domain linear fitting, the raw weighted fused spectral centroid already exhibited a consistent monotonic relationship with the applied preload.

**Figure 9:**
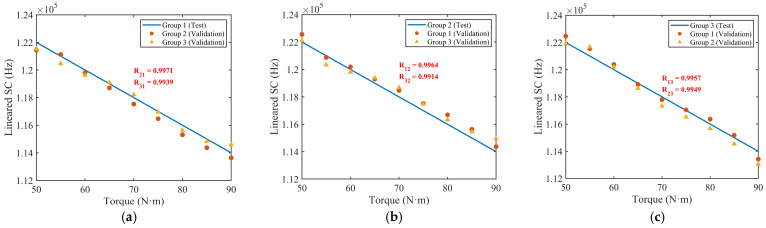
Reference-group-based validation results of the spectral centroid fusion strategy: (**a**) 1st loading group used for calibration; (**b**) 2nd loading group used for calibration; (**c**) 3rd loading group used for calibration.

The fixed SC-domain linear relationship was then used to express the validation responses in the linearly fitted fused-spectral centroid domain. Figure 9 presents the relationship between the fused spectral centroid response and bolt preload across the three loading experiments under different reference-group selections. All validation groups achieved Pearson correlation coefficients greater than 0.99, indicating high linear consistency and experimental repeatability under the present repeated loading validation protocol. Because the fusion weights and SC-domain linear calibration coefficients were fixed after reference-group fitting, the validation groups did not contribute to weight optimization or coefficient fitting.

Table 3 summarizes the validation metrics obtained under different reference-group selections. In the validation groups, the fused spectral centroid index maintained a highly consistent monotonic relationship with the applied preload. Because the Pearson correlation coefficient R is invariant under fixed linear calibration, the reported R values also characterize the pre-calibration linear association between the raw weighted fused spectral centroid and the applied torque. The $RMSE_{SC}$ and $MaxE_{SC}$ values in Table 3 were calculated from the residuals between the observed fused spectral centroid values and the corresponding linearly fitted fused spectral centroid values; therefore, they are reported in Hz. These values describe the index-domain residual deviation of the fused spectral centroid index across repeated loading groups and provide an indication of its repeatability. From the perspective of preload monitoring, the consistently high R values and limited SC-domain residuals indicate that the fused spectral centroid index maintained a stable monotonic relationship with the applied torque within the investigated range of 50–90 N·m.

These results indicate that the proposed multi-frequency spectral centroid fusion strategy enables stable calibration transferability and good repeatability under the investigated tightly coupled single-bolt condition.

### 4.3. Influence of Excitation Conditions and Structural Parameters on Spectral Centroid

In the robustness analyses involving variations in excitation amplitude, structural length, and temperature, the arithmetic mean of the spectral centroids was used as the global response feature. This parameter-free formulation was selected to assess the intrinsic stability of the spectral centroid descriptor across multiple reconstructed frequency bands without introducing preload-optimized weights. By contrast, the linear weighted fusion strategy described in Section 4.2 was optimized for preload identification using a reference loading group, and the resulting weights are therefore specific to the preload calibration task. Applying these preload-oriented weights directly to the disturbance datasets could couple the effects of preload calibration and environmental perturbations, thereby complicating the interpretation of feature robustness. The following disturbance analysis focuses on the feature-level stability of the spectral centroid descriptors before preload calibration.

The spectral centroid metric used in this section represents the arithmetic mean of spectral centroids from 14 narrowband reconstructed signals with distinct center frequencies. This simplification facilitates fusion computation while evaluating overall stability under parameter perturbations.

**Figure 10:**
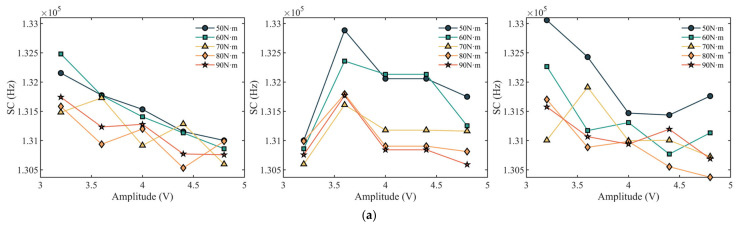

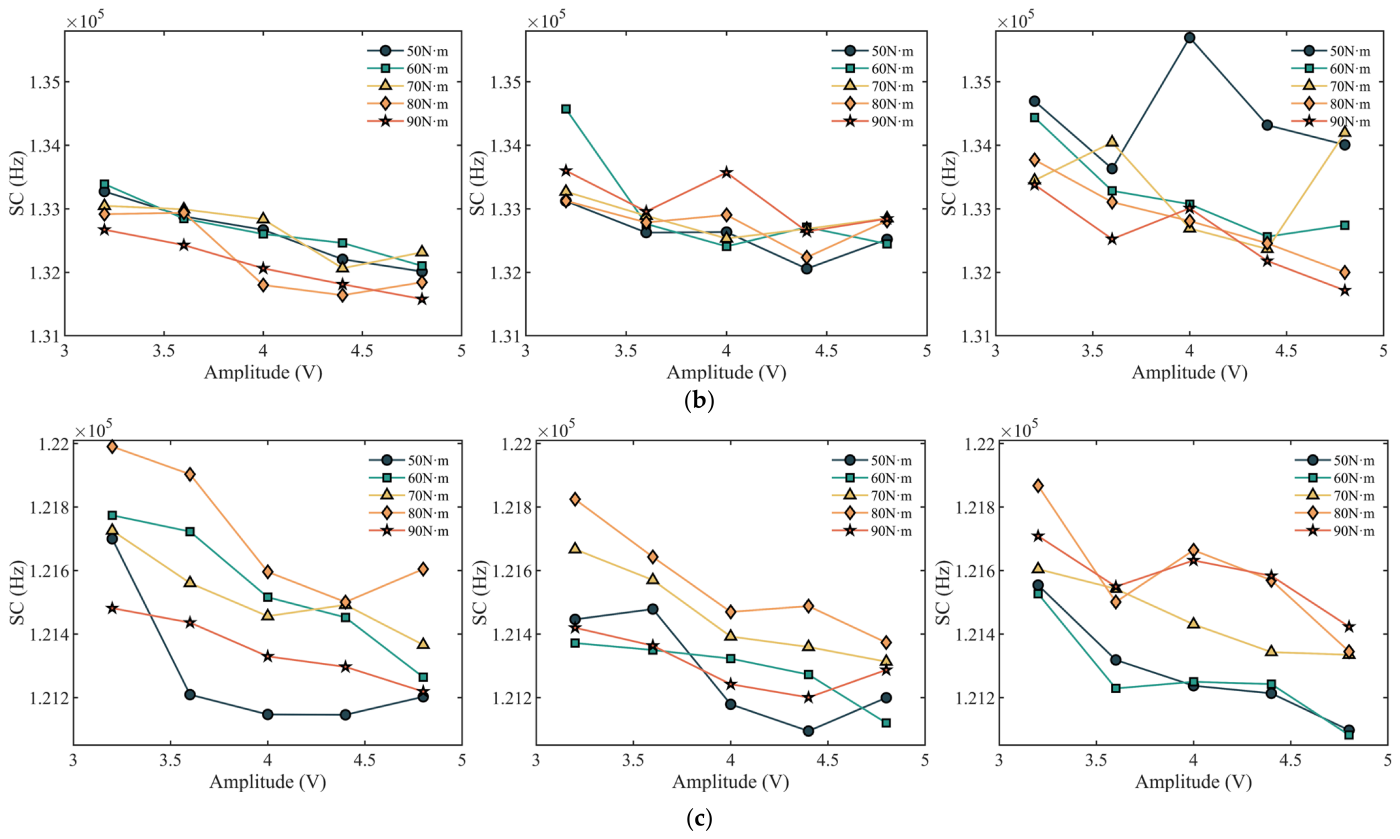
Spectral centroid variation under different excitation amplitudes and torques for steel plates of different lengths: (**a**) 180 mm; (**b**) 225 mm; (**c**) 300 mm.

To analyze the impact of excitation amplitude and structural parameters on spectral centroids, responses were examined under three steel plate lengths (180 mm, 225 mm, and 300 mm) and varying excitation amplitudes (3.0–5.0 V). Figure 10 and Table 4 show the variation trends of spectral centroid features under these conditions, with the key findings summarized as follows:

(1) Stability Performance: Across all plate dimensions, spectral centroids exhibit minimal variation (<3%) with changing excitation voltage. At the same time, curves remain closely clustered without systematic drift, demonstrating excellent normalization properties and robustness consistent with theoretical expectations;
(2) Adaptability Performance: Despite differences in plate length, the arithmetic mean spectral centroid maintains a similar response morphology under the tested loading states. The relative range remains below 3.0% for all tested plate lengths, with values of 2.02%, 2.93%, and 0.64% for the 180 mm, 225 mm, and 300 mm plates, respectively. These results indicate that the spectral centroid descriptor exhibits limited variation under the tested structural length and excitation amplitude conditions.

In summary, the arithmetic mean spectral centroid showed limited variation under changes in excitation amplitude and structural length. For the structural length analysis, the recalculated relative ranges were 2.02%, 2.93%, and 0.64% for the 180 mm, 225 mm, and 300 mm plates, respectively, all of which remained below 3.0%. These results support the feature-level stability of spectral-centroid-based descriptors under the tested disturbance conditions. However, because the weighted fusion model was calibrated specifically for preload identification, its overall robustness under simultaneous variations in preload, excitation amplitude, structural dimensions, and environmental conditions should be further evaluated using a larger factorial experimental design.

### 4.4. Analysis of Temperature Variation Effects

To evaluate the temperature sensitivity of spectral features, temperature perturbation tests were conducted under constant excitation at three preload levels: 50, 60, and 70 N·m. The arithmetic mean of the spectral centroids across the reconstructed frequency bands was used as the global response feature for comparative analysis. This parameter-free setting was adopted to examine the intrinsic temperature stability of the spectral centroid descriptor without introducing preload-optimized weights. This section evaluates the feature-level robustness of the spectral centroid descriptors under temperature variations.

**Figure 11:**
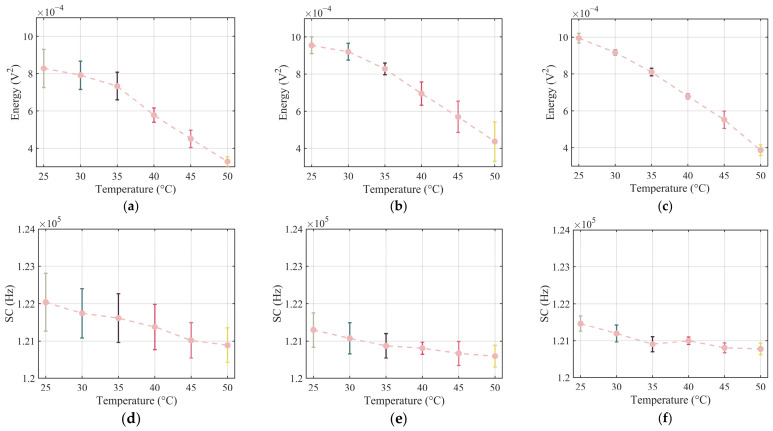
Variation in time-domain energy (**a**–**c**) and spectral centroid (**d**–**f**) with temperature: (**a**,**d**) 50 N·m; (**b**,**e**) 60 N·m; (**c**,**f**) 70 N·m.

Figure 11 illustrates the trends of spectral centroids and energy features versus temperature:

(1) Energy Feature Behavior: Exhibits a significant decrease with rising temperature, accompanied by larger fluctuations. Additionally, the curves demonstrate pronounced attenuation, confirming high sensitivity to environmental disturbances and poor stability;
(2) Spectral Centroid Behavior: Shows gradual linear variation with temperature increase, and the curves remain stable with low dispersion across samples, indicating strong trend consistency and repeatability.

To accurately quantify the impact of temperature variations on time-domain energy and frequency-domain spectral centroid features, the following metrics are employed for evaluation: range percentage (Range%) and relative standard deviation (RSD). The results are presented in Table 5.

(17) $$\mathrm{Range}\%=\frac{\max\left(x\right)-\min\left(x\right)}{\max\left(x\right)}\times 100\%$$

where $max\left(x\right)$ = maximum value in the dataset, and $min\left(x\right)$ = minimum value in the dataset. Interpretation: *Range%* quantifies the normalized spread between extreme values relative to the maximum value. A higher Range% indicates greater amplitude variation in the feature. The maximum value was used as the normalization reference because *Range%* was designed to quantify the largest relative spread of a feature within the tested temperature interval under the same preload condition. This definition provides a dimensionless measure that enables direct comparison between energy-based and spectral centroid features, despite their different units and magnitudes.

(18) $$RSD=\frac{\sigma }{\mu }\times 100\%$$

where μ = the mean value of the dataset, and σ = the standard deviation of the dataset. Interpretation: The *RSD* measures relative volatility. A smaller *RSD* implies lower dispersion and higher stability; conversely, a larger *RSD* signifies inconsistent or unstable data.

The results in Table 5 demonstrate the following:

(1) At all preload levels, the *Range%* and *RSD* values of the spectral centroid are substantially lower than those of the energy-based feature. Specifically, the Range% reductions reach 98.42%, 98.93%, and 99.08% at 50, 60, and 70 N·m, respectively;
(2) For the *RSD*, the corresponding reductions are 98.89%, 99.21%, and 99.31%, respectively. These results indicate that the arithmetic mean spectral centroid exhibits substantially lower temperature-induced fluctuation than the energy-based feature under the tested preload conditions.

In summary, the arithmetic mean spectral centroid produced more stable trend outputs than the energy-based feature under temperature disturbances. As shown in Table 5, the spectral centroid reduced *Range%* by 98.42–99.08% and the *RSD* by 98.89–99.31% relative to the energy-based feature. This improvement can be mainly attributed to the normalization term in the spectral centroid calculation. Energy-based features are directly influenced by amplitude attenuation, whereas the spectral centroid characterizes the redistribution of spectral energy and is therefore less sensitive to uniform amplitude variations. These results demonstrate the feature-level environmental stability of spectral-centroid-based descriptors within the proposed framework.

## 5. Conclusions

This study proposes a guided-wave-based method that integrates multi-frequency narrowband pulse reconstruction with spectral centroid fusion to identify bolt preload through calibration transfer within a controlled single-bolt laboratory configuration. The main conclusions are as follows:

(1) Multi-Frequency Reconstruction: Frequency-domain filters constructed from Chirp sweep responses enable the reconstruction of narrowband guided wave signals at multiple center frequencies. This provides multidimensional information for frequency feature extraction, reduces excitation cycles, and enhances detection efficiency;
(2) Weighted Fusion Strategy: The proposed spectral centroid fusion strategy improves the linear correlation with preload by optimizing the multi-frequency fusion weights using the reference loading group and then applying an SC-domain linear calibration relationship. Across repeated loading validation groups, the fused features achieved Pearson correlation coefficient R values exceeding 0.99, indicating a high degree of linear consistency and calibration transfer repeatability;
(3) Robustness of Spectral Centroid Features: Compared with conventional energy-based metrics, the arithmetic mean spectral centroid exhibited greater feature-level stability under variations in excitation amplitude, structural length, and temperature. In the temperature experiments, the arithmetic mean spectral centroid reduced *Range%* by 98.42–99.08% and the *RSD* by 98.89–99.31% relative to the energy-based features. These results suggest the intrinsic robustness of the spectral centroid descriptor adopted in the proposed framework. However, the environmental robustness of the final calibrated weighted-fusion estimator should be further assessed using expanded datasets that simultaneously account for variations in preload, temperature, structural dimensions, and excitation conditions;
(4) Generality and Extensibility: The present study demonstrates the calibration transferability and repeatability of the weighted-fusion framework under repeated loading within the tested single-bolt configuration. The excitation amplitude, temperature, and limited plate length experiments provide feature-level evidence for the stability of spectral-centroid-based descriptors. However, the environmental validation of the full calibrated weighted-fusion preload estimator under combined operating variations remains a subject for future work.

Although validated for single-bolt structures under static loads, practical engineering challenges remain. Future work should explore applications in multi-bolt structures, complex damage identification, and intelligent feature fusion.

For engineering applications, the proposed method indicates a potential offline calibration–online monitoring framework for the tested single-bolt configuration. During baseline calibration, performed during installation or scheduled maintenance, the fusion weights and global SC-domain linear calibration coefficients are determined using reference measurements. Once this fixed model has been established, changes in the fused spectral centroid response can be tracked for condition monitoring within the calibrated configuration. The extension of this framework to field deployment requires further validation under sensor reinstallation, structural variability, dynamic loading, and combined environmental disturbances.

The fusion weights and SC-domain linear calibration coefficients are determined from a reference loading group and then kept fixed for all validation groups. During validation, the measured guided wave responses are first transformed into fused spectral centroid features using the fixed weight vector derived from the reference group. The resulting fused spectral centroid values are then evaluated using the fixed SC-domain linear calibration relationship, with no preload-dependent correction or scaling factor introduced. This validation setting prevents information leakage from the validation data and frames the present study as a proof-of-concept validation on a single bolted connection under static preload conditions. Consequently, the results mainly demonstrate calibration transferability and repeatability within the investigated preload range of 50–90 N·m for the same bolted-joint configuration. Accordingly, the identification claims are limited to the controlled single-bolt laboratory configuration and this preload interval. Although this interval is relevant for the early warning of preload loss in the present specimen, further validation is required before the method can be extended to unseen torque levels, wider preload ranges, different bolt diameters, different plate thicknesses, sensor reinstallation conditions, dynamic service loads, and multi-bolt connection structures. In addition, the disturbance experiments assessed the robustness of spectral-centroid-based descriptors using the arithmetic mean formulation. Because these tests provide feature-level evidence rather than direct validation of the calibrated weighted-fusion estimator, the environmental robustness of the final weighted-fusion preload estimator should be further verified using larger datasets covering combined operating conditions.

Extension of the proposed method to multi-bolt joints requires further investigation. In such structures, guided wave propagation can be affected by scattering from multiple fasteners, overlapping reflection paths, crosstalk among adjacent bolt interfaces, and more complex boundary conditions. As a result, the fusion weights calibrated for the current single-bolt configuration cannot be directly applied to arbitrary multi-bolt structures without additional calibration or spatial decoupling. The present calibration procedure adopts a fixed model form. Specifically, the fusion weights and SC-domain linear calibration coefficients are determined using only the reference loading group and are subsequently applied unchanged to all validation samples. The validation results are evaluated based on the monotonicity, linear consistency, and SC-domain residuals of the observable fused spectral centroid response. Accordingly, the current results should be regarded as proof-of-concept evidence of calibration transferability and repeatability within the tested single-bolt configuration. Future work will focus on multi-bolt connection scenarios, sensor reinstallation effects, and combined environmental disturbances. These issues will be addressed by integrating the proposed spectral centroid fusion framework with sensor arrays, path selection strategies, and imaging-based localization methods.

## Figures and Tables

**Table 1:** Parameters of the sweep excitation signal.

Start Frequency	Step Size	Stop Frequency	Amplitude	Duration
20 kHz	1 kHz	250 kHz	4 V	0.231 s

**Table 2:** Waveform similarity, Pearson correlation coefficient, and normalized mean square error between the reconstructed signal and the signal actually received.

Frequency (kHz)	Waveform Similarity (%)	Correlation Coefficient (%)	NMSE (%)
50	97.51	97.52	4.91
80	99.82	99.82	0.43
100	99.83	99.83	0.35
120	99.88	99.88	0.27
150	99.86	99.86	0.28
180	99.82	99.82	0.37

**Table 3:** Validation results for the fixed multi-frequency fusion model and SC-domain linear calibration model across repeated loading groups.

Reference Group	Validation Group	$RMSE_{SC}$ Linearly Fitted Fused-SC Residuals (Hz)	R	$MaxE_{SC}$ Linearly Fitted Fused-SC Residuals (Hz)
1st Loading	2nd Loading 3rd Loading	488.4 395.5	0.9971 0.9939	741.2 612.0
2nd Loading	1st Loading 3rd Loading	490.9 541.4	0.9964 0.9914	746.0 950.8
3rd Loading	1st Loading 2nd Loading	396.3 539.5	0.9957 0.9949	615.1 943.0

**Table 4:** Relative range of spectral centroid for steel plates of different lengths under different excitation amplitudes and torques.

Length (mm)	SC_max_ (kHz)	SC_min_ (kHz)	Relative Range (%)
180	133.06	130.37	2.02
225	135.69	131.72	2.93
300	121.87	121.08	0.64

**Table 5:** Relative variation in time-domain energy and frequency-domain spectral centroid characteristic quantities under the same preload and different temperature conditions.

Preload (N·m)	Energy (V^2^)		Spectral Centroid (Hz)	
	Range (%)	RSD (%)	Range (%)	RSD (%)
50	60.23	32.37	0.95	0.36
60	54.30	27.90	0.58	0.22
70	61.09	31.74	0.56	0.22

## Data Availability

The data presented in this study are available on request from the corresponding author.

## Abbreviations

The following abbreviations are used in this manuscript:

SC	Spectral Centroid
NMSE	Normalized Mean Square Error
CAN	Contact Acoustic Nonlinearity
CNN	Convolutional Neural Network
DAQ	Data Acquisition
FFT	Fast Fourier Transform
GW	Guided Wave
SNR	Signal-to-Noise Ratio
LTI	Linear Time-Invariant
MaxE	Maximum Error
VAM	Vibro-Acoustic Modulation
PZT	Lead Zirconate Titanate
R	Pearson Correlation Coefficient
RMSE	Root Mean Square Error
RSD	Relative Standard Deviation
